# Women’s experiences and health care-seeking practices in relation to uterine prolapse in a hill district of Nepal

**DOI:** 10.1186/1472-6874-14-20

**Published:** 2014-02-03

**Authors:** Binjwala Shrestha, Sharad Onta, Bishnu Choulagai, Amod Poudyal, Durga Prasad Pahari, Aruna Uprety, Max Petzold, Alexandra Krettek

**Affiliations:** 1 Department of Community Medicine and Public Health, Institute of Medicine, Tribhuvan University, Kathmandu, Nepal; 2 Department of Internal Medicine and Clinical Nutrition, Institute of Medicine, Sahlgrenska Academy at University of Gothenburg, Gothenburg, Sweden; 3 Rural Health and Education Trust, Kathmandu, Nepal; 4 Akademistatistik- Centre for Applied Biostatistics, Occupational and Environmental Medicine, University of Gothenburg, Gothenburg, Sweden; 5 Nordic School of Public Health NHV, Gothenburg, Sweden

**Keywords:** Uterine prolapse, Health seeking practice, Nepal

## Abstract

**Background:**

Although uterine prolapse (UP) occurs commonly in Nepal, little is known about the physical health and care-seeking practices of women with UP. This study aimed to explore women’s experiences of UP and its effect on daily life, its perceived causes, and health care-seeking practices.

**Methods:**

Using a convenience sampling method, we conducted 115 semi-structured and 16 in-depth interviews with UP-affected women during September–December 2012. All interviews occurred in outreach clinics in villages of the Dhading district.

**Results:**

Study participants were 23–82 years of age. Twenty-four percent were literate, 47.2% had experienced a teenage pregnancy, and 29% had autonomy to make healthcare decisions. Most participants (>85%) described the major physical discomforts of UP as difficulty with walking, standing, working, sitting, and lifting. They also reported urinary incontinence (68%) bowel symptoms (42%), and difficulty with sexual activity (73.9%). Due to inability to perform household chores or fulfill their husband’s sexual desires, participants endured humiliation, harassment, and torture by their husbands and other family members, causing severe emotional stress. Following disclosure of UP, 24% of spouses remarried and 6% separated from the marital relationship. Women perceived the causes of UP as unsafe childbirth, heavy work during the postpartum period, and gender discrimination. Prior to visiting these camps some women (42%) hid UP for more than 10 years. Almost half (48%) of participants sought no health care; 42% ingested a herb and ate nutritious food. Perceived barriers to accessing health care included shame (48%) and feeling that care was unnecessary (12.5%). Multiple responses (29%) included shame, inability to share, male service provider, fear of stigma and discrimination, and perceiving UP as normal for childbearing women.

**Conclusions:**

UP adversely affects women’s daily life and negatively influences their physical, mental, and social well-being. The results of our study are useful to generate information on UP symptoms and female health care seeking practices. Our findings can be helpful for effective development of UP awareness programs to increase service utilization at early stages of UP and thereby might contribute to both primary and secondary prevention of UP.

## Background

Uterine prolapse (UP), also known as pelvic organ prolapse or genital prolapse, is a reproductive health problem [1]. In this condition, failure of ligamentous and fascial supports causes the uterus to descend into or beyond the vagina, resulting in protrusion of the vagina, the uterus, or both [2]. Uterine displacement into the vagina is usually graded 0–3 (or 4), with 0 referring to no prolapse and 3 (or 4) referring to total prolapse (procidentia) [3]. Common symptoms include feeling or seeing a vaginal bulge [4]. UP affects the health and social well-being of women, particularly in the reproductive and economically productive age group [5].

The mean prevalence of pelvic organ prolapse in low- and middle-income countries is 19.7% (range 3.4%–56.4%) [6]. In Nepal, UP prevalence varies in different ecological zones, from 20%–37% in the Terai (Plain) area [7] and 25% in the far west hills [8] to 27.4% in the central and eastern hills [9]. Nationally, UP prevalence is 7%–10% [10,11].

In Nepal, the main symptoms of UP are difficulty with lifting, sitting, walking (80%–89%); urinary problems (30.7%); and painful intercourse (41.1%). Additional complaints include backache; abdominal pain; burning on urination; white, watery discharge; foul-smelling discharge; and itching (27%–55%) [8].

A review of evidence in Nepal points to a significant decline in the maternal mortality ratio from 415 deaths per 100,000 live births in 2001 to 281 in 2006 [12]. However, despite progress in maternal health care UP remains persistent in all districts of both urban and rural communities and therefore poses a public health problem in Nepal.

Although the districts are gradually implementing a UP policy, women are not motivated to use such services. Female community health volunteers bridge the distance from households to health facilities and outreach clinics. Barriers to medical help for UP include women’s reluctance to seek treatment due to lack of family support; ineffective treatment; and high cost for travel, food, and lodging [13]. Discriminatory gender norms and value systems make women more vulnerable to gender-power relations and place men in a higher position. The entire society considers men’s dominant behavior as normal [14].

Nepal’s traditional gender divisions of labor encourage women to concentrate more on their reproductive roles and household responsibilities (mostly unpaid), whereas men work outside the home because most are educated and free to move for work [7].

Women with UP might present with vague symptoms which poses difficulties for proper diagnosis. Additionally, current knowledge about women’s UP symptoms and experience, how UP influences their daily lives and their health care-seeking practices regarding UP is sparse. Our study is therefore timely and relevant as it contributes to increased knowledge about women’s UP symptoms. We here describe women’s experiences of UP and its effects on daily life, its perceived causes, and women’s health care-seeking practices in a rural hilly district of Nepal. The results of our study are useful to generate information on UP symptoms and female health care seeking practices. Our findings can be helpful for effective development of UP awareness programs to increase service utilization at early stages of UP and thereby might contribute to both primary and secondary prevention of UP.

## Methods

### Study setting

Our study was conducted in nine hilly rural village development committees (VDCs) in the Dhading district of Nepal during September–December 2012. The 2011 Census of Nepal listed the total population of Dhading district as 338,658 and average family size was 5.4. The adult literacy rate was 43.7% (34% and 53.9% for women and men, respectively). The contraceptive prevalence rate was 24.4%. Women’s and men’s age at first marriage is 16.41 and 21.96 years, respectively [15]. In Nepal’s human development index, Dhading ranked 55^th^ among 75 districts. Life expectancy at birth was 58.55 years and the gender development index was 0.394 [16]. Most of the population works in subsistent agriculture and has low income for basic livelihood. Transportation facilities in these villages are limited due to harsh terrain [17].

UP management camps were organized in Dhading from 3–12 April 2012. The camps, which aimed to provide pessary treatment for women with UP, identify UP incidence, and refer women for surgical treatment, counseled and assisted more than 954 women from Dhola (144), Nalang (178), Salang (137), Fulkharka (121), Salyantar (140) and Jyamrung (234) [18]. Follow-up outreach clinics for women diagnosed with UP were organized during September–December 2012. Follow-up clinics also diagnosed new cases. Medical teams for clinical services comprised of gynecologists, international volunteers, and nurses as well as resident doctors from the district hospital in Dhading.

### Study design

**Figure 1:**
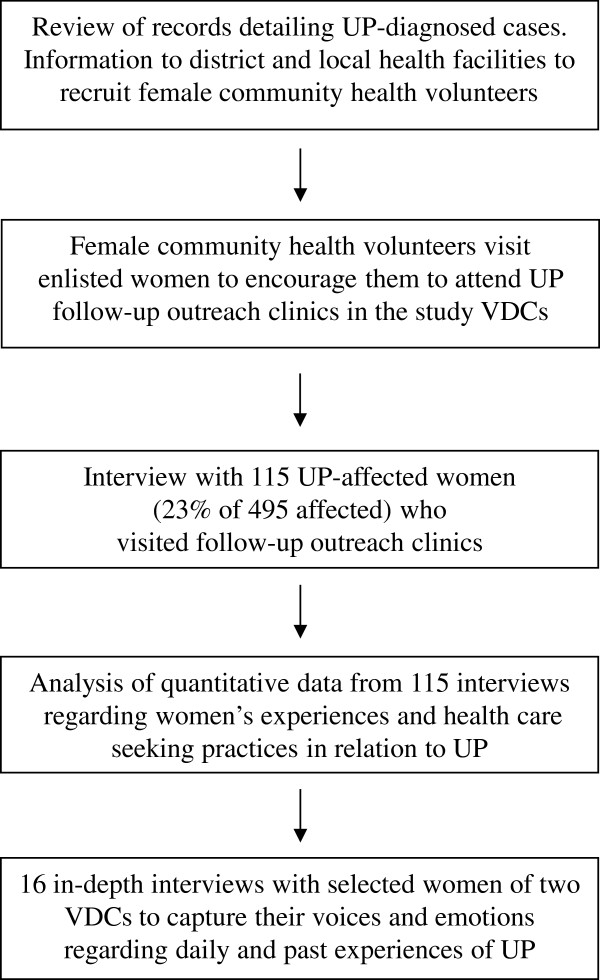
**Outline of the research process.** We used a mixed methods approach to explore women’s experiences and health care-seeking practices in relation to uterine prolapse (UP) in nine hilly rural village development committees (VDCs) in the Dhading district of Nepal. Both quantitative and qualitative data were collected at the same stage of the research process. The process is outlined in sequence to demonstrate how both the quantitative and qualitative studies were organized.

This descriptive and explorative study employed a pragmatic framework using mixed methods [19]. We collected both quantitative and qualitative data at the same stage of the research process [20]. The research process is outlined in Figure 1.

### Study participants

For the quantitative study, participants were included based on convenience sampling at the follow-up outreach clinics. We did not use control groups for comparison. All participants had been diagnosed by a gynecologist and trained medical officer with first-, second-, or third-stage UP according to scoring in the pelvic organ prolapse questionnaire [21]. We visited nine follow-up outreach clinics during the research period. A total of 495 UP-affected women visited the clinics, yielding an average of 55 women per clinic. Among those, we interviewed 23% (N = 115) of UP-affected women using a simple random sample method. No one refused to be interviewed.

The inclusion criteria for in-depth interviews included (i) first-time UP diagnosis in an outreach clinic, to explore experiences during the journey to reach the outreach camp; (ii) long history of untreated UP, to explore reasons why women delay care-seeking practice; (iii) early reproductive age (i.e., the 20–30 years age group), to explore specific experiences of young women; iv) UP with severe urinary incontinence, to explore complications from UP; and (v) women from poor and marginalized groups, to explore care-seeking practices and barriers.

### Data collection

We reviewed the records of previous UP screening camps and identified women who required follow-up. Then we coordinated with the district health office and health workers of local health facilities in our study VDCs to mobilize female community health volunteers. The female community health volunteers visited the houses of the women who, according to our list, were already diagnosed with or had symptoms of UP and encouraged them to attend follow-up outreach clinics. These were located in health facilities of the study VDCs such as Aganchok, Chainpur, Dhola, Fulkharka, Jyamrung, Salyangkot, Salyangtar, Tripureswar, Salang, and other VDCs. Prior to the interview at the follow-up outreach clinic, each participant was individually informed about the objective of the study and was ensured privacy and confidentiality. We also explained that their participation would be useful for developing tools for early diagnosis of UP and to facilitate the design of health messages for health communication programs targeting UP.

### Quantitative data collection

For quantitative data collection, two trained female researchers, one graduate nurse and one sociologist used pre-tested, semi-structured questionnaires to interview participants at outreach clinics.

### Qualitative data collection

We collected qualitative data through in-depth interviews (conducted by the first author) with selected participants who had specific experiences of UP. Respondents were informed about the purpose of the study before the interviews were conducted. Altogether, we conducted in-depth interviews with 16 UP-affected women in follow-up outreach clinics organized in Salyantar and Jamrung VDCs on 3–4 December 2012. We used an audio recorder to capture the women’s voices, and we stopped the interview when we achieved saturation.

### Data analysis

#### Variables and quantitative data analysis

We reviewed main variables of interest and divided them into 10 areas: (i) socioeconomic characteristics (i.e., age, education, occupation caste/ethnicity, and income); (ii) reproductive characteristics of women and stages of UP; (iii) decision making for health care; (iv) experiences of physical discomfort; (v) UP-related sexual discomfort; (vi) emotional stress, gender discrimination, and domestic violence; (vii) spousal behavior after disclosure of UP; (viii) experiences of domestic violence following disclosure of UP; (ix) self-perceived reasons for UP; and (x) health care-seeking practices for UP. We categorized variables regarding experiences of UP according to a study on experiences of genital prolapse [22]. Data were processed in SPSS version 17 (SPSS Inc., Chicago, Illinois, USA).

#### Qualitative data analysis

**Figure 2:**
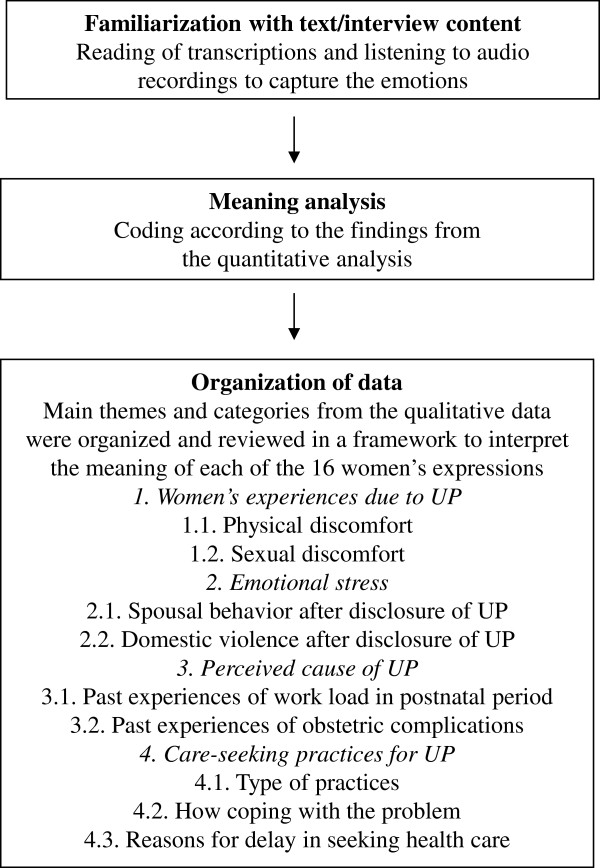
**Process of qualitative data analysis.** The process of qualitative data analysis is outlined in chronological order. We used a deductive approach for content analysis of in-depth interviews. Main themes and categories were mainly based on issues identified in the quantitative study.

We used a deductive approach for content analysis of our in-depth interviews [23]. Main themes and categories were mainly based on issues identified in the quantitative study. The process involved familiarization with the material, identifying a thematic framework, indexing, charting, mapping and interpretation [24]. Briefly, we used an audio recorder to capture participants’ voices and transcribed the recordings verbatim. To familiarize ourselves with the meaning of participants’ descriptions, we listened to the audiotapes of each in-depth interview. Next, we reviewed the content of individual transcriptions to identify themes for analysis. The process of qualitative data analysis as well as four main themes and individual categories are outlined in Figure 2.

Next, we reviewed quantitative and qualitative findings and organized them under the same domain/variable of analysis. Finally, we interpreted the data and described both quantitative and qualitative findings in an integrated way.

### Ethical considerations

UP is still taboo in the Nepalese society. Because women fear that others will know their problems, they might be reluctant to share personal information with others. They also might fear the consequences of sharing personal experiences and exposing themselves to potential harm from their spouse and other family members. Therefore, we explained these issues to each participant and conducted interviews only after receiving informed consent. Study participants had autonomy to respond to each question; we also discussed and ensured both anonymity and confidentiality prior to each interview. This study was approved by the Nepal Health Research Council (Reg. no. 56/2012) and interviews were conducted according to the national guidelines of the Nepal Health Research Council.

## Results

### Socioeconomic characteristics of participants

A total of 115 UP affected women were from 10 VDCs of the Dhading district, namely Aganchok (10.43%), Chainpur (6.96%), Dhola (6.96%), Fulkharka (11.30%), Jyamrung (16.52%), Saklyangkot (8.70%), Salyangtar (9.57%), Tripureswar (10.43%), Salang (8.70%) and other nearby villages (10.43%).

A majority of women (67%) were from an advantaged ethnic group (i.e., Brahmin, Chhetri, or Newar). Socially marginalized (i.e., disadvantaged) women from the Janajati and Dalit groups accounted for 11% and 19% of our study population, respectively. More than 95% of participants were Hindu. Sources of income included agriculture (56%), services (14%), business and labor (6%), foreign remittance (5%), and multiple sources including agriculture and business (12%). Average family size was seven and in most households (59%) men were the main decision makers. Participants’ ages ranged from 23 to 82 years (mean age 54 ± 15 years); 82% were married and currently living with spouse, 11% were widows, and 6% were separated. Women’s mean age at marriage was 16 years; 25% were literate, compared to 63% of men (spouse). Eighty-four percent of women’s main occupations were farming or household chores (Table 1).

**Table 1:** Distribution of socioeconomic characteristics of study participants

**Variables**	**N**	**%**
**Caste/ethnic group**		
Advantaged group (Bramhin, Chhetri, and Newar)	77	67.0
Disadvantaged group (Tamang, Rai, hill Janajati)	13	11.3
Disadvantaged and socially suppressed group (Dalit)	22	19.1
Others	3	2.6
**Main source of income**		
Farming	65	56.5
Service	16	13.9
Business	7	6.0
Labor	7	6.0
Foreign remittance	6	5.2
Multiple sources	14	12.1
**Sufficiency of income in a year**		
3 months	8	6.9
6 months	48	41.7
> 6 months	57	49.5
> 1 year	2	1.7
**Age group (years)**		
23-35	14	12.1
36-50	38	33.0
51-60	25	21.7
61-70	21	18.2
≥ 71	17	14.7

Results are shown for 115 women interviewed in nine rural hilly districts of Nepal while attending uterine prolapse follow-up outreach clinics.

### Reproductive characteristics of women and stages of UP

The average number of pregnancies per woman was nine, and 47% (n = 107) had experienced pregnancy during her teen-age years. The average number of live births was seven, the average number of miscarriages was four. Among those diagnosed with UP, 11% had first stage, 60% second stage, and 25% third stage. Three percent of participants had vault prolapse.

### Experiences of physical discomfort

Many participants had self-diagnosed UP before examination by clinical experts. Participants experienced multiple physical discomforts and emotional stress due to UP and physical discomforts often accompanied vaginal symptoms. Most (>85%) participants described a bulging sensation and a heavy feeling in their vagina due to UP. Affected women also reported difficulty walking, sitting, doing manual work, etc. This influenced their daily work by limiting their mobility and capacity for manual work in the household and the fields (Table 2). Sixty-eight percent of participants said they suffered from urinary incontinence. Among these, 59% also experienced burning and pain during urination and 28% experienced stress incontinence during coughing and sneezing. Urinary symptoms made it difficult to maintain hygiene and women were embarrassed to face family members and friends (Table 2). Some women suffered from urinary incontinence for long durations, resulting in severely impaired quality of life that led to other reproductive health problems. A 55-year-old said:

*I was suffering from dripping urine with bad smell for 16 years with occasional vaginal bleeding*.

**Table 2:** Distribution of uterine prolapse symptoms according to experiences by women in follow-up outreach clinics

**Symptoms**	**N**	**%**
**Vaginal problems**		
Bulging of uterus and closure of vaginal opening	13	11.3
Difficulty sitting, standing, lifting, back pain, lower abdomen pain, prolonged bleeding	18	15.7
Difficulty standing, sitting, walking, lifting, bleeding	37	32.1
Difficulty sitting, standing, walking, lifting, Back pain, bleeding	47	40.9
**Urinary incontinence**		
Burning and pain during urination	46	59.0
Urination while coughing and sneezing	22	28.2
Continuous urinary incontinence	10	12.8
**Bowel problems**		
Multiple problems; need to reduce and hold uterus during defecation (digitization)	32	71.0
Constipation and pain during defecation	9	20.0
Fecal leakages	4	8.8

Forty-two percent of participants reported suffering from various types of bowel symptoms and 71% had multiple symptoms, including the need to reduce and hold the uterus manually during defecation, constipation with painful defecation, and fecal leakage (Table 2). During an in-depth interview, a 68-year-old woman said as:

*I had difficulty on defecation; the pessary will come out while defecating so I need to hold the pessary and I am frequently suffering from diarrhea and constipation but have not done health check up till now*.

### Sexual discomfort

Most participants (74%) mentioned that they discontinued sexual activities. They gave various reasons for discontinuing sexual activities, including (i) no sexual desire due to old age (36%); (ii) pain and difficulties during sexual intercourse (7%); (iii) conflict and separation with spouse (13%); and (iv) multiple reasons, including conflict with spouse, and fear of spousal awareness of UP (29%).

The remaining affected participants (26%) said they had periodic sexual relationships with their spouse. Among those, one fourth experienced painful sexual intercourse. During an in-depth interview, one 50-year-old participant said:

I don’t have any sexual desire it is painful but I must fulfill my husband’s desire, can’t get any support from my husband. I am forced to tolerate the painful sexual relation…

### Spousal behavior after disclosure of UP

A majority (60%) of spouses of affected women who had disclosed UP were not bothered by the diagnosis. Twenty-three percent of spouses were caring and supportive, but 16% harassed their wives by threatening to remarry or separate. Twenty-four percent of spouses remarried after their wives disclosed a UP diagnosis.

### Domestic violence after disclosure of UP

About 50% of participants did not wish to respond to questions relating to experiences of violence. Among those who responded, 30.4% reported that they had experienced domestic violence after disclosing UP to their spouse. Additionally, in-laws humiliated and harassed them because they could not perform the expected level of household work. Participants also reported that they had experienced violence including physical assault and verbal abuse. Specific violence included heavy workload, threat of remarriage, decreased amounts of food, and physical abuse. During an in-depth interview, one 55-year-old woman said:

Before operation I was facing various problems from my family. My sister-in-law used to scold me and insulted me by using abusing words because I was incapable to do household chores. I was excluded in many social functions because I could not maintain hygiene. Many times my sister-in-law scolded me by saying “leave home”; I was about to leave home but the neighbor rescued me. Now after operation, I am also harassed by my husband and sister-in-law because my working capacity is still not restored.

### Self-perceived reasons of UP

Self-perceived reasons for UP included repeated pregnancies and childbirth (40%), lack of rest during the postnatal period (31.3%), and heavy lifting in daily housework (18%). Among affected women, 77% resumed household chores within 6–20 days of delivery, 13% resumed work within 1–5 days, and 8% within 21–30 days.

During in-depth interviews, participants mentioned that they did not have opportunity to rest because, according to tradition, the daughter-in-law is responsible for all household chores. Daily chores included carrying water, caring for cattle, cleaning, washing clothes, cooking, doing agricultural work, etc. Some women did not receive adequate food even though they continued to do their daily household chores throughout pregnancy. A 27-year-old woman said:

My mother died and I could not go to my maternal house. I was striving for food; there was no one to support me. I used to carry heavy load (water, fodder), do regular household activities, I did not get chance to take rest. I resumed household work within 11 days of delivery.

Similarly, 37% of participants had experienced obstetric complications such as postpartum hemorrhage and obstetric labor that lasted more than two days. About 19% of participants faced both types of obstetric complications.

During in-depth interviews, almost all women said that they delivered children at home. Some were referred to a health facility or called a health worker if they encountered complications. Most had faced multiple obstetric complications, including prolonged labor (more than 5 days) and heavy bleeding, with or without retained placenta. A 66-year-old woman described her past experience as

During first child delivery, I faced 6 days and nights of labor pain and at that time everything was gone (total tear of pelvic floor). Then during second child delivery the placenta was retained and at that time also I was about to die. Again on third time delivery (latest) I could not work for six months, my youngest daughter cared for me.

### Health care-seeking practices for UP

Mean age at first perception of UP was 34 ± 10 years and mean duration of suffering from UP was 23 ± 15 years. Among study participants, 23% immediately communicated their UP problem to their relatives or health workers. However, 35% hid their UP problem for more than 10 years. Prior to visiting the outreach clinics, participants had not visited health facilities for various reasons, including shame (48%), not felt as necessary (12%), and multiple responses (29%), including shame, having no one to share about the problem, male service provider, fear of stigma and discrimination, and a perception that UP is normal for childbearing women, etc. One 49-year-old woman expressed her feelings and described how she was coping with UP as:

I feel something coming out and block the opening of the vagina. I need to replace it manually. The severity increases while carrying loads (water, fodder, firewood etc.), washing, cleaning, walking and standing and sitting in squatter position.

Affected women practiced various modes of self-care before visiting the outreach clinic. Forty-eight percent were coping by reducing the uterus manually, 9% consulted local health workers, and 42.5% used multiple practices, such as Bethi (a herb that resembles a green vegetable) and eating nutritious food (e.g., meat, eggs, milk, fruits, etc.).

After intervention at an outreach clinic, participants began using health services as instructed by experts. Today, they regularly visit a local health facility and an outreach clinic for follow-up treatment. Among 115 women, 62% used a pessary, 13% had surgical treatment, and 20% used pelvic exercise and herbal medication. Clinical records indicate that surgical services mostly involved vaginal hysterectomy at referral hospitals in Kathmandu.

During in-depth interviews, participants further explained their reasons for using services in the follow-up outreach clinics and health facilities. The most pertinent symptom for seeking health care was urinary incontinence. One 40-year-old woman reported:

I am using pessary since 4 years. The doctor recommended surgical treatment, there is free treatment. However, I have to manage travel and other additional costs for patient visit and food. I cannot afford these additional costs. My son is not taking care of me; maybe God will take care of me….

## Discussion

Uterine prolapse is a public health problem in Nepal that permeates society at all levels [10]. Despite UP being a common condition, there is limited knowledge on specific symptoms and women’s perceptions [8]. Our study explored women’s UP-related daily life experiences, their perceptions regarding treatment as well as barriers for seeking health care.

### Physical discomfort and quality of life

Women’s physical discomfort varied according to the severity and duration of exposure and treatment/non treatment. Despite such discomfort, they continued to do their household chores and gradually developed more symptoms that eventually rendered them incapable of contributing to household chores. The consequences of UP for women’s physical and sexual health, working ability, and earning capacity are extensive [14]. UP damage to the pelvic muscles, which then become unable to close the vaginal opening. Because too much force can break any structure, muscles and ligaments may fail when stressed beyond their limits. Obesity or working in a job that requires repeated heavy lifting may expose the pelvic floor to unusual pressure, which might explain why some women with pre-existing susceptibility to UP experience damage to the pelvic floor. Moreover, the same factors may provoke UP [25].

### Emotional stress due to gender-based violence

Emotional stress links to emotional abuse, which occurs commonly with other types of gynecological problems. Severe physical symptoms result in low desire for sexual intercourse as well as urinary incontinence, which often restricts women’s social lives [22]. Study participants exhibited low self-esteem, which they frequently described as anxiety, aggression, frustration, and despondency. Affected women guiltily accepted any undesired sexual relationship and endured painful sexual intercourse because they depend on their spouse’s income. We found that most spouses are not interested in engaging with their wives after they disclose UP. Moreover, spouses and other family members humiliated and harassed women with UP. Men believed that UP is an inevitable result of childbirth and obstetric complications. In another study, men reported that UP increases women’s vulnerability to violence and stigmatization by their spouses [26]. Moreover, UP greatly impairs a woman’s ability to work, which directly affects their family’s assessment of them [8].

### Gender discrimination and health care-seeking practices

According to the Nepal Demographic and Health Survey 2011, only 40% of Nepalese women are illiterate and 61% of working women are not paid for their work. In contrast, only 14% men are illiterate and 76% earn cash or cash and in-kind payments for their work [12]. However, in our study area most women had low socioeconomic background with 75% being illiterate and 56% having either farming or unpaid jobs. The literacy rate for women and men was 25% and 63%, respectively. This substantial educational gap impacts women’s employment and decision making opportunities regarding their health.

Gender bias regarding education and choice of occupation opportunities influences women’s low social and economic position, increasing their dependence on spouses or other family members, possibly contributing to a delay in seeking health care for UP treatment. Nepal has a patriarchal society and women are expected to conduct domestic chores, do planting, collect fodder and take care of livestock. In most families in both rural and urban areas, men are the decision makers and even dominate women’s reproduction and labor access; women are treated as subordinate [27].

Gender inequality is a key factor that underlies several determinants of UP in Nepal. Women are facing barriers to access health care for UP such as lack of awareness about available treatment, lack of decision making authority for medical treatment, and lack of funds to cover treatment costs. It is challenging even for educated women to seek and access health care due to fear of divorce or abandonment, isolation and the sensitivity surrounding genital issues [28].

### Perceived causes of UP and health care-seeking practices

Participants in our study suggested that the reasons for UP were unsafe delivery practices, obstetric complications, and heavy workload during the postnatal period. This could link with their experiences of obstetric complications, risky home delivery practices, and postnatal workload in previous delivery practices. Like most women in Nepal, our participants perceived that they are responsible for all household chores, even during pregnancy and the postnatal period. Consequently, women felt they could not demand health care during early-stage UP. However, other studies have suggested that UP results from carrying heavy loads every day and tightening the abdominal musculature, thereby increasing pressure on the pelvic organs [3,8].

### Cultural issue and delay in health care-seeking

We determined that one perceived barrier to health care for UP was a woman’s feeling of shame, because UP involves a sensitive part of the body. Thus, a “culture of silence” and “laaj” (i.e., shame about reproductive health) restricts women from talking about pregnancy and its related problems [29]. This is a major contributor to the frequent failure of women and families to recognize and acknowledge the significance of symptoms that indicate possible complications during pregnancy and birth, consequently delaying a decision to seek help [22]. Because women fear condemnation by their community and their families, they mostly hide their UP problem and continue their silence [30].

The socioeconomic characteristics, reproductive behavior, symptoms of UP and its effects on daily life as well as health care seeking practices reported here may not reflect all women suffering from UP across the diversity of socio-economic strata. Future population-based studies should therefore include respondents from all socio-economic strata to allow for increased generalizability of the findings. Despite these limitations, our study has a number of strengths. We have examined women’s health related quality of life and care practices and access issues in the context of women’s lives which might enable early identification and management of potential health risks which may remain into older age.

## Conclusions

UP adversely affects women’s daily life and negatively influences physical, mental, and social well-being. The results of our study are useful to generate information on UP symptoms and female health care seeking practices. These findings can be helpful for effective development of UP awareness programs to increase service utilization at early stages of UP and thereby might contribute to both primary and secondary prevention of UP.

## Competing interests

The authors declare that they have no competing interests.

## Authors’ contributions

BS designed the study, developed the data collection tools, undertook data analysis, and drafted the manuscript. SO and AK contributed to structure and content of the paper. DP supervised and aided in data collection. BC gave input on an early manuscript draft. AP and MP provided input on quantitative data analysis, AU commented on the discussion of the manuscript. All authors have read and approved the final manuscript.

## Pre-publication history

The pre-publication history for this paper can be accessed here:

http://www.biomedcentral.com/1472-6874/14/20/prepub
